# Recurrent Multifocal Embolic Strokes in a 50-Year-Old Male: Unmasking Occult Squamous Cell Carcinoma

**DOI:** 10.7759/cureus.45091

**Published:** 2023-09-12

**Authors:** Deipthan Prabakar, Vaishnavi Sabesan, Oluwasegun P Emenogu, Cuc Mai

**Affiliations:** 1 Internal Medicine, University of South Florida Morsani College of Medicine, Tampa, USA

**Keywords:** embolic stroke, multifocal infarctions, occult malignancy, cancer-related stroke, recurrent stroke, cryptogenic

## Abstract

Recurrent cryptogenic embolic strokes pose a diagnostic challenge, often necessitating an extensive evaluation to determine the underlying cause. Cancer-related stroke is a frequently overlooked etiology, accounting for a substantial proportion of cryptogenic strokes. This case study underscores the importance of considering occult malignancies in patients with recurrent strokes of unknown origin and emphasizes the need for a comprehensive diagnostic workup to detect hidden malignancies.

A 50-year-old male with a complex medical history presented with expressive aphasia and blurred vision resembling previous stroke episodes. Neurological examinations revealed right hemianopsia, paraphasia, and abnormal coordination. Neuroimaging studies showed multiple chronic infarctions, a large hemorrhagic infarction in the left posterior cerebral artery territory, and a small acute-to-subacute left parietal infarction. Due to the time of presentation and the presence of hemorrhagic transformation, the patient did not meet the criteria for intravenous tissue plasminogen activator administration. Given the recurrent nature of the strokes, an extensive evaluation was initiated to determine the underlying cause. Vascular imaging, including magnetic resonance angiography (MRA) of the head and neck and a CT angiogram, showed no significant stenosis. Vasculitis workup and cardiac evaluation yielded negative results. The blood workup was notable for elevated D-dimer levels. The involvement of multiple vascular territories and recurrent stroke despite adequate treatment and the absence of traditional risk factors for stroke raised a high clinical suspicion of occult malignancy. Further investigations led to the diagnosis of locally advanced squamous cell carcinoma (P16+), metastatic to the right neck lymph nodes (cTxN3M0). Although the primary source of cancer could not be identified, the P16+ status suggests the right tonsil or base of the tongue as the probable origin. Anticoagulation therapy was initiated, and the patient was scheduled for chemoradiation therapy.

Although routine cancer investigation is not justified in ischemic strokes, the possibility of an occult malignancy should be considered in the presence of multifocal infarctions across different vascular territories with elevated D-dimer levels, particularly when traditional risk factors have been ruled out. A detailed physical exam can help localize the malignancy and early identification of occult malignancies can guide appropriate management strategies and help prevent future strokes. Further clinical trials are needed to establish optimal therapeutic approaches for preventing stroke recurrence in cancer-related strokes.

## Introduction

Ischemic strokes are a major cause of disability and mortality globally, necessitating a comprehensive evaluation to determine their underlying etiology [1]. Recurrent strokes present a diagnostic challenge, particularly when conventional risk factors and embolic sources have been addressed.

## Case presentation

A 50-year-old male patient, with a medical history of coronary artery disease, hypertension, type 2 diabetes mellitus (A1C 6.1), asthma, obstructive sleep apnea (compliant with continuous positive airway pressure (CPAP)), chronic kidney disease (baseline creatinine of 3.4), and thrombotic thrombocytopenic purpura treated with rituximab, as well as a history of drug-eluting stent placement for coronary artery disease and previous strokes in 7/2022 and 12/2022, presented to the emergency department. Additionally, he was a current smoker.

He came to the hospital because he had trouble finding words and experienced blurred vision. He described waking up feeling normal until around 11 a.m. when he began to feel disoriented and had difficulty finding words. Upon returning home, his wife noticed his slurred speech, which resembled his previous stroke symptoms. Over the past week, he had been experiencing visual problems, and his ophthalmologist suspected these issues were due to transient ischemic attacks that had affected his right visual field. The patient also mentioned experiencing chronic headaches but denied having fevers, chest pain, or shortness of breath.

Two weeks before this admission, he had a transesophageal echocardiogram that identified a small patent foramen ovale and an implantable loop recorder was placed. Additionally, he had a heart catheterization performed six months ago, which, according to both the patient and his wife, did not reveal any significant issues.

In the emergency department, the patient was hemodynamically stable with BP 128/83 mmHg, pulse 82/min, temperature 97.7 F, respiratory rate 20/min, and oxygen saturation (SpO2) 96%. The patient was alert and oriented with normal attention and concentration. Cardiovascular and respiratory examinations were unremarkable. A thorough neurological exam was performed, which revealed right hemianopsia and paraphasia (substitution of words during speech) with a National Institutes of Health Stroke Scale (NIHSS) score of 3. Except for visual field testing, cranial nerve examination showed no abnormalities. The patient had a motor strength of 4/5 in the right upper extremity and 5/5 in the remaining extremities. The coordination test was abnormal, and the gait was ataxic. Tone, reflexes, and sensations were normal. Pertinent lab findings include a low hematocrit of 38.6%, elevated troponin of 0.140 ng/mL (with repeat troponin trending at 0.140, likely secondary to demand ischemia in the setting of stroke), creatinine of 3.1 mg/dL, blood urea nitrogen (BUN) of 28 mg/dL, and total protein of 8.4 g/dL (Tables 1-3).

**Table 1:** Complete blood count WBC: White Blood Cell Count, RBC: Red Blood Cell Count, MCV: Mean Corpuscular Volume, MCH: Mean Corpuscular Hemoglobin, MCHC: Mean Corpuscular Hemoglobin Concentration, MPV: Mean Platelet Volume, RDW: Red Cell Distribution Width

Parameter	Value	Reference Range (Males)
WBC	13.37 x 10^9 cells/L	4.5-11.0 x 10^9 cells/L
RBC	4.79 x 10^12 cells/L	4.3-5.8 x 10^12 cells/L
Hemoglobin	11.0 g/dL	13.8-17.2 g/dL
Hematocrit	38.6%	38.8-50.0%
MCV	91.0 fL	80-100 fL
MCH	29.4 pg	27-33 pg
MCHC	32.3 g/dL	32-36 g/dL
Platelet Count	236 x 10^9 cells/L	150-450 x 10^9 cells/L
MPV	11.5 fL	7.4-10.4 fL
RDW	13.7%	11.5-14.5%
% Neutrophils	66.3%	40-75%
% Immature Granulocytes	0.6%	0-1.5%
% Lymphocytes	18%	20-40%
% Monocytes	11.2%	2-10%
% Eosinophils	3%	0-6%

**Table 2:** Complete metabolic profile BUN: Blood Urea Nitrogen, AST: Aspartate Aminotransferase, ALT: Alanine Aminotransferase, ALP: Alkaline Phosphatase, GFR: Glomerular Filtration Rate

Parameter	Value	Reference Range
Sodium	138 mmol/L	135-145 mmol/L
Potassium	3.6 mmol/L	3.5-5.0 mmol/L
Chloride	108 mmol/L	98-106 mmol/L
Bicarbonate	21 mmol/L	22-28 mmol/L
Glucose	186 mg/dL	140-200 mg/dL
BUN	28 mg/dL	7-20 mg/dL
Creatinine	3.1 mg/dL	0.6-1.3 mg/dL
Calcium	10.0 mg/dL	8.5-10.5 mg/dL
Total Protein	8.4 g/dL	6.4-8.3 g/dL
Albumin	3.9 g/dL	3.5-5.0 g/dL
Globulin	5 g/dL	2.3-3.5 g/dL
Albumin/Globulin Ratio	1	1.2-2.2
Total Bilirubin	0.3 mg/dL	0.1-1.2 mg/dL
ALP	93 U/L	30-120 U/L
AST	12 U/L	5-40 U/L
ALT	13 U/L	7-56 U/L
Anion Gap	12 mEq/L	8-16 mEq/L
BUN/Creatinine Ratio	9	10-20
GFR	24 mL/min/1.73m²	Normal GFR > 60 mL/min/1.73m²

**Table 3:** Other Investigations TSH: Thyroid-Stimulating Hormone, HDL: High-Density Lipoprotein, LDL: Low-Density Lipoprotein, HbA1C: Hemoglobin A1C

Parameter	Value	Reference Range
D-dimer	5.2 mg/L	0-0.50 mg/L
TSH	4 mIU/L	0.4-4.0 mIU/L
Total Cholesterol	162 mg/dL	<200 mg/dL
Triglycerides	118 mg/dL	<150 mg/dL
LDL	89 mg/dL	<100 mg/dL
HDL	50 mg/dL	>40 mg/dL
HbA1C	6.6%	<5.7%

**Figure 1:**
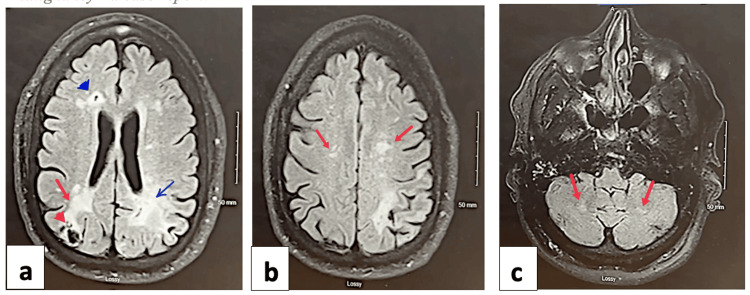
MRI brain – T2 FLAIR axial a) Infarctions in B/L posterior cerebral artery territory R - stable (blue arrow), L - chronic (red arrow) but new from the previous study in a large portion of the left occipital lobe with prior hemorrhagic transformation(red arrowhead); small, chronic, but new inferior frontal gyrus cortical infarction (blue arrowhead); b) B/L chronic unchanged parietal infarctions;(red arrows); c) old lacunar infarctions in cerebellar hemispheres (red arrows)

The CT scan of the head showed no acute hemorrhage. Vascular neurology and cardiology were consulted, and a loop recorder interrogation was performed. Due to the elevated creatinine, a CT angiogram was deferred. To further investigate the recurrent strokes, an immediate MRI of the brain and MRA head and neck was ordered. The MRI brain (Figure 1) revealed multiple chronic infarctions in both the supratentorial and infratentorial brain regions, including a large hemorrhagic infarction in the left posterior cerebral artery territory, which was new compared to the previous MRI. An additional small acute to subacute infarction was detected in the left parietal region. The patient did not meet the criteria for IV tissue plasminogen activator administration due to the time of presentation being outside the treatment window and the presence of hemorrhagic transformation in the recent infarction. Continuous telemetry monitoring and neurochecks every four hours were initiated.

A comprehensive evaluation was conducted to determine the underlying cause of the recurrent strokes. Vasculitis workup, including treponemal antibody testing, yielded negative results. A hematologist/oncologist was consulted. Potential conditions included emboli from occult or paroxysmal atrial fibrillation, paradoxical emboli through a patent foramen ovale, hypercoagulable conditions, and internal carotid artery stenosis.

Blood pressure, low-density lipoprotein (LDL), and A1C were within the normal range. The EKG showed sinus rhythm without T wave inversions or ST changes. Aspirin reaction units and P2Y12 reaction units were within the therapeutic range. The implantable loop recorder (ILR) interrogated by cardiology showed no evidence of occult/paroxysmal atrial fibrillation. Echo with bubble study revealed a normal ejection fraction with no atrial shunts. Venous ultrasound of the lower extremities showed no evidence of deep vein thrombosis. MRA of the head and neck revealed no significant stenosis. The blood workup was notable for an elevated D-dimer level. The presence of multiple infarcts on diffusion-weighted imaging involving different vascular territories, especially in regions adjacent to major blood vessels, along with an unremarkable cardiac evaluation, raised suspicions of occult malignancies.

**Figure 2:**
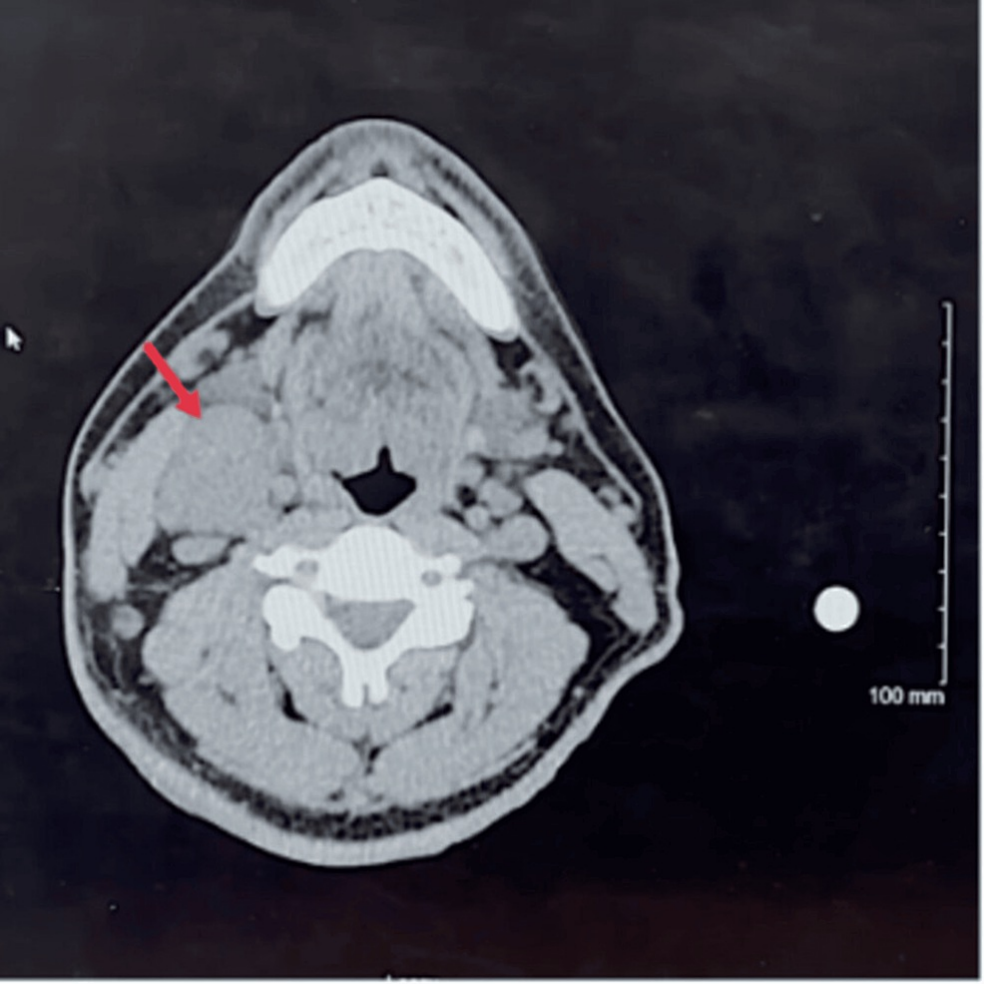
CT soft tissue neck CT soft tissue neck demonstrating a right neck soft tissue mass measuring approximately 3.8 x 2.7 x 6.0 cm.

Physical examination revealed palpable nodes on the right side of the neck, with one measuring 1.5 cm in diameter and the other measuring 1 cm in diameter. A review of the MRA of the neck images revealed a mass in the right neck. Subsequently, a CT soft tissue neck scan was performed, confirming the presence of a right neck soft tissue mass measuring approximately 3.8 x 2.7 x 6.0 cm (Figure 2).

A core needle biopsy of the right neck mass confirmed it as metastatic squamous cell carcinoma (P16+). However, the primary source of cancer could not be identified on positron emission tomography (PET)/CT or nasopharyngolaryngoscopy. Nevertheless, due to the P16+ status, it most likely originates from either the right tonsil or the base of the tongue. CT imaging of the chest, abdomen, and pelvis showed no significant findings, and the PET/CT scan identified a hypermetabolic mass in the right neck at cervical lymph node levels 2 and 3, with no other hypermetabolic lymphadenopathy or abnormal metabolism detected in the chest, abdomen, or pelvis. Due to the potential risks associated with holding anticoagulation, pan endoscopy was deferred, as it was unlikely to change management. The patient was diagnosed with locally advanced squamous cell carcinoma (P16+), metastatic to the right neck lymph nodes (cTxN3M0), and is planned for chemoradiation therapy.

## Discussion

According to the TOAST classification (Trial of Org 10172 in Acute Stroke Treatment - (a classification system for ischemic stroke)) (Table 4) [1], cryptogenic stroke refers to a brain infarction without a definite source, such as cardio-embolism, large artery atherosclerosis, or small artery disease, despite thorough evaluation [2]. Although classic stroke etiologies are common in cancer patients, cryptogenic stroke is more frequent and strongly associated with cancer [3]. The prevalence of arterial embolic events in cancer patients is as high as 4.7% [4]. Approximately 20% of patients with strokes of undetermined cause have an occult malignancy at the time of presentation [5]. Studies show that a stroke can be the initial sign of cancer, with 84% of these patients experiencing a second stroke within four months [6].

**Table 4:** TOAST classification of subtypes of acute ischemic stroke Source: [1] TOAST: Trial of Org 10172 in Acute Stroke Treatment (a classification system for ischemic stroke)

Subtypes
Large-artery atherosclerosis
Cardio embolism
Small-vessel occlusion
Stroke of other determined etiology
Stroke of undetermined etiology

Common cancer types associated with recurrent stroke include breast, gynecological, cholangiocarcinoma, lung, pancreatic, gastric, and metastatic cancer [6]. This is a rare case report demonstrating squamous cell carcinoma of the head/neck as a cause of multifocal embolic stroke.

Embolic stroke patterns offer valuable insights into the source, with single vascular territory involvement pointing to a proximal large artery (carotid or vertebrobasilar) origin, whereas sources proximal to that have the potential to distribute emboli throughout all vascular territories [7]. The presence of micro-embolic scattering on diffusion-weighted (DW) MRI in multiple vascular territories may serve as a potential indicator of underlying cancer in stroke patients [8].

Among the various mechanisms of malignancy leading to stroke (Table 5), cancer-induced hypercoagulability can lead to thromboembolism through the interaction of cytokines, tissue factors, cancer procoagulants, and mucin [9]. These factors accelerate the coagulation cascade and promote platelet activation, resulting in the dissemination of thromboembolism. In stroke, this hypercoagulability can cause emboli formation through paradoxical embolization, clot aggregates on cardiac valves, and hypercoagulability due to secreted substances [10]. The finding of negative transthoracic echocardiography, negative Holter monitoring, and clear angiography, along with an elevated D-dimer in this case, argues for cryptogenic stroke due to hypercoagulability.

**Table 5:** Mechanisms underlying cancer-related stroke Source: [3,11]

Mechanisms
Cerebral intravascular coagulation due to increased expression of procoagulant factors
Cerebral micro-emboli from nonbacterial thrombotic endocarditis
Thoracic radiation-induced aortic atheroma
Anthracycline/ trastuzumab/ radiation-induced cardiomyopathy
Paradoxical embolus from venous thromboembolism
Infective endocarditis/sepsis due to an indwelling catheter
Conventional stroke due to shared risk factors
Infections
Direct tumor effect

D-dimer levels, which serve as a biomarker for hypercoagulability and short-term stroke risk in cancer patients, have shown higher levels in patients with cryptogenic stroke and occult cancers compared to those without cancer [12]. Gou et al. demonstrated that high D-dimer levels (≥1.55 mg/l) were present in the majority of cancer patients with acute ischemic stroke, compared to those without cancer [13]. Combining D-dimer (≥0.55 mg/l) with multiple territory infarctions increased specificity to 99.7% but had low sensitivity (24.5%). A cutoff of ≥5.5 mg/l provided high specificity (99.6%) irrespective of multiple territory infarctions, suggesting its potential for malignancy detection in stroke patients. However, the predictive utility of D-dimer levels can be influenced by various factors such as tumor burden, age, and diabetes, complicating its interpretation [6].

Clinical trials and guidelines primarily focus on cancer-associated venous thrombosis [14], and the application of these findings to arterial thrombotic events like ischemic strokes remains uncertain. Controversies exist regarding the initiation and choice of anticoagulation in active malignancy. For cryptogenic stroke, the initial treatment approach aligns with other ischemic strokes, including intravenous thrombolysis with alteplase and mechanical thrombectomy [11]. Active cancer should not be viewed as an absolute contraindication to tissue plasminogen activator use [11]. However, cancer patients with clot-related occlusions often have a higher fibrin/platelet composition, multiple vessel involvement, and challenging recanalization requiring diverse techniques and multiple attempts [15].

Apart from managing predisposing factors, antiplatelet therapy, and statins for secondary prevention, It has been proposed that for individuals with a documented history of cancer and unexplained recurrent embolic events affecting the brain and/or various organs, venous thromboembolism (VTE), or the diagnosis of adenocarcinoma may belong to a category prone to hypercoagulability. Therefore, in the presence of any of these factors, it is advisable to consider anticoagulant treatment using low molecular weight heparin (LMWH) as it may offer potential benefits [3]. Direct oral anticoagulants (DOACs) are an attractive option due to their convenience and wider therapeutic window. Studies show comparable efficacy and safety between DOACs and low molecular weight heparin for treating cryptogenic ischemic stroke in active cancer patients, with potential benefits for those with high D-dimer levels [16,17]. Due to potential drug interactions between anticoagulants and chemotherapeutic medications, which are often caused by the CYP3A4 enzyme and P-glycoprotein transporter, caution is necessary while prescribing DOACs, and this risk is even higher in cases of renal insufficiency, which is common among cancer patients [14]. Combining anticoagulants and antiplatelet drugs may reduce the thrombotic burden and recurrent stroke risk as seen in the COMPASS (Cardiovascular Outcomes for People Using Anticoagulation Strategies) trial [18]. Administering anticoagulants to cancer patients with elevated D-dimer levels may decrease arterial infarction events, but the decision should consider the higher bleeding risk [19]. Currently, there are no established regulations for the treatment of CRS, and large-scale randomized prospective studies are required to establish specific diagnostic and treatment algorithms for stroke in cancer patients.

Ischemic strokes are often caused by identifiable medical factors like cardioembolism, large vessel atherosclerosis, or small vessel disease [20]. If a patient shows infarction in multiple vascular territories on MRI and has elevated D-dimer levels, despite negative findings in standard stroke evaluations and appropriate treatment, it is essential to look for possible malignancy-associated thrombophilia. A comprehensive physical examination helps us narrow down the potential sites of malignancy. Patients with cancer who experience a stroke are at a high risk of short-term stroke recurrence and have a poor prognosis. In patients diagnosed with cancer, the cumulative recurrence rate of stroke after one month of the first episode is 7%, and the recurrence rate at six months reaches 16% [19]. This experience underscores the need for vigilant monitoring and preventive measures in this high-risk population. Identifying the underlying pathology promptly is crucial for prognostic purposes and to guide appropriate investigations and management strategies.

## Conclusions

In summary, this case report underscores the complex connection between cryptogenic stroke and hidden malignancy. It highlights the need for thorough evaluation when conventional stroke risk factors fall short. The mechanisms of cancer-induced hypercoagulability, evidenced by elevated D-dimer levels, emphasize the importance of understanding the underlying processes. While treatment approaches align with standard stroke care, the challenges posed by concurrent malignancy warrant tailored strategies, including the potential use of DOACs. This case underscores the ongoing need for research and refined guidelines to navigate the intricate interplay between stroke and cancer, enabling timely detection and optimized treatment outcomes for patients.
